# High-frequency oscillatory ventilation in adults: experience in Chile

**DOI:** 10.1186/cc9613

**Published:** 2011-03-11

**Authors:** SU Ugarte, CR Rojas, C Herrera

**Affiliations:** 1 Clinica INDISA, Santiago, Chile

## Introduction

The aim was to describe the epidemiological profile of adult patients who were treated with HFOV like a rescue method after conventional mechanical ventilation failure, during 2009 in our ICU, in Santiago, Chile, and to describe patient characteristics, HFOV strategies and outcomes.

## Methods

A descriptive study. We evaluated the medical record of all adult patients treated with HFOV during 2009 at Clínica INDISA. We evaluated sex, age, associated co-morbidities, laboratory test results and main diagnosis at ICU admission, hours in conventional mechanical ventilation previous to HFOV connection, indication of HFOV, laboratory test results at the connection time to HFOV, and patient outcome.

## Results

A total of 15 patients were treated with HFOV during 2009 in our ICU; the mean age was 47 years, being 80% men. Three patients did not have, at ICU admission or during the course of the current hospitalization, description of associated co-morbidities, while 53.3% had report of two or more co-morbidities. The main diagnosis at ICU admission was severe pneumonia (53.3%) with a mean APACHE II score of 27.7. The mean values for PaFi and IOX prior to HFOV connection were 108.8 and 25, respectively. The main indication observed in those patients was very high FiO_2 _requirement to achieve an adequate arterial oxygen saturation (60% of the cases). Twenty percent of the sample required reconnection to HFOV, the mortality in this group of patients was 100%. Of all patients that were exposed to HFOV, there was an effective weaning to CMV and medical discharge in 40% of them, while the mortality during HFOV was 60%.

## Conclusions

We present the epidemiological profile of the patients exposed to HFOV during 2009 at our medical center, the mean age at admission was 47 years old; the main diagnosis was severe pneumonia, 40% of all patients survived. HFOV has beneficial effects on PaO_2_/FiO_2 _ratios and OI, and may be an effective rescue therapy for adults with severe oxygenation failure. This is the first study of its kind at a national level.
